# Computation Emerges from Adaptive Synchronization of Networking Neurons

**DOI:** 10.1371/journal.pone.0026467

**Published:** 2011-11-04

**Authors:** Massimiliano Zanin, Francisco Del Pozo, Stefano Boccaletti

**Affiliations:** 1 Centre for Biomedical Technology, Polytechnic University of Madrid, Pozuelo de Alarcón, Madrid, Spain; 2 Innaxis Foundation & Research Institute, Madrid, Spain; Tel Aviv University, Israel

## Abstract

The activity of networking neurons is largely characterized by the alternation of synchronous and asynchronous spiking sequences. One of the most relevant challenges that scientists are facing today is, then, relating that evidence with the fundamental mechanisms through which the brain computes and processes information, as well as with the arousal (or progress) of a number of neurological illnesses. In other words, the problem is how to associate an organized dynamics of interacting neural assemblies to a computational task. Here we show that computation can be seen as a feature emerging from the collective dynamics of an ensemble of networking neurons, which interact by means of adaptive dynamical connections. Namely, by associating logical states to synchronous neuron's dynamics, we show how the usual Boolean logics can be fully recovered, and a universal Turing machine can be constructed. Furthermore, we show that, besides the static binary gates, a wider class of logical operations can be efficiently constructed as the fundamental computational elements interact within an adaptive network, each operation being represented by a specific motif. Our approach qualitatively differs from the past attempts to encode information and compute with complex systems, where computation was instead the consequence of the application of control loops enforcing a desired state into the specific system's dynamics. Being the result of an emergent process, the computation mechanism here described is not limited to a binary Boolean logic, but it can involve a much larger number of states. As such, our results can enlighten new concepts for the understanding of the real computing processes taking place in the brain.

## Introduction

Synchronization is one of the most important features observed in neural systems [1]–[3], and it certainly is the basic mechanism for the processing and integration of information across functionally and anatomically specialized regions of the brain [4]–[6]. The current understanding is that neural assemblies are the basic computation units, composed of networks of neurons, which intermittently share information, and transiently use dynamical connections. However, synchronization alone is clearly not enough to ensure a computational capability, which requires, instead, a proper balance between synchronous and asynchronous behavior [7]. A modification of this balance is related to a number of neurological illnesses, including schizophrenia [8] and Alzheimer [9]. A complete understanding of how synchronization is related to computation is still lacking, and synchrony is sometimes even seen as a source of knowledge destruction, as a number of different systems collapse into a single, shared dynamics.

Here we show that computation can emerge from the synchronization of groups of adaptively coupled neurons. Such collective dynamics can encode information within different synchronization states, and efficiently perform any Boolean operations, thus being able to construct a universal Turing machine [10]. While our study is in the spirit of past attempts focused on defining computation outside the digital realm, which made use of chaotic systems to manipulate information [11]–[14], the main message here is that computation arises as an emergent feature of synchronization, and it does not need additional external control loops such as thresholds [12], [13] or specific initial conditions [14].

## Methods

**Figure 1:**
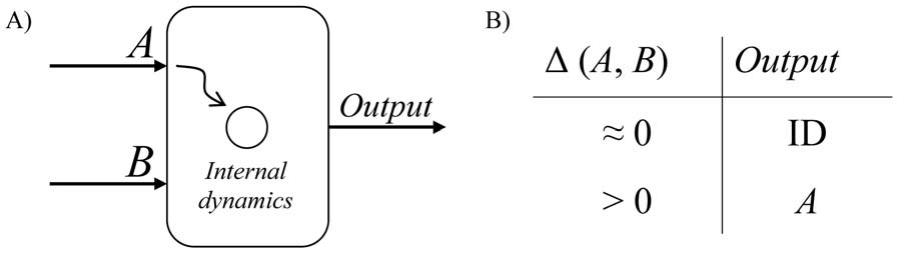
Basic computation unit. A) Schematic representation of the basic computation unit. A neuron, subject to a common Gaussian noise, receives two control signals from ports *A* and *B*. B) Output signal of the neuron as a function of 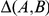 (the synchronization error between the signals entering ports *A* and *B*). If 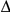 is of the order of 0 (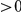), the neuron's output sequence of spikes follows the internal dynamics *ID* (the spike sequence of the signal entering from port *A*).

The basic computational unit (see Figure 1a) is here constituted by: i) a neuron, modeled by the Hodgkin-Huxley equations [15], ii) two input ports (*A* and *B*), and iii) one output port. The Hodgkin-Huxley model of neurons describes changes in the membrane potential 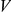 by the equation:
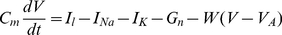
(1)where 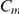 is the membrane capacitance and 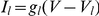 a passive leak current. We assume that an ensemble of such neurons is networking, so that each neuron is forced by the external signal 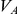 entering from port *A*, with a coupling strength *W* defined by an adaptive coupling mechanism (see Figure 1a). Furthermore, all neurons are under the effect of a common external source of Gaussian noise 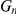, so that, in the absence of external forcing or coupling, their autonomous dynamics results in a specific synchronous spike pattern *ID* [16], [17]. Following the original model introduced by Hodgkin and Huxley [15], 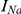 and 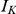 represent simplified de- and repolarizing (respectively, Na and K) currents. The current for the Na ion is defined as follows:
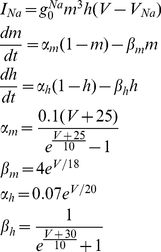
(2)


The K channel is modeled by the following set of equations:
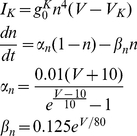
(3)


Constants are common for all neurons, and their values are 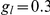, 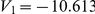, 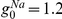, 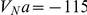, 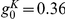 and 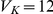. The integration is performed using the first order Euler method, with *dt* equal to 0.005.

We further assume that at least one reference spike pattern exists (henceforth called *R*), that is generated by units that are not under the influence of the same Gaussian noise, and is therefore not synchronous with the common internal dynamics *ID*. Each neuron is coupled to an input signal that enters from port *A*, whose coupling strength *W* is the result of the following adaptive dynamics:
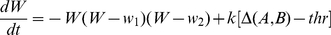
(4)


In this equation, 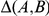 represents the synchronization error between signals entering ports *A* and *B*, that is, the proportions of spikes in the pattern entering from port *A* (*B*) which do not correspond to a spike in the signal of port *B* (*A*). When 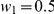 and 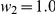, the first term of the rhs of Eq. 4 creates three equilibrium points, two of them stable (zero and one). The second term forces the system toward one of these two equilibrium points, depending on the synchronization error 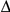 between the spike patterns entering from ports *A* and *B*. 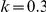 is a positive parameter that defines the velocity of adaptation, and 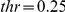 is a threshold, used to filter small synchronization errors that may arise from random sources of noise. In summary, the adaptive dynamics is defined in such a way that the coupling strength *W* tends to zero when the synchronization error between the input signals entering ports *A* and *B* is vanishing, i.e., when both inputs are synchronized, and to a positive value otherwise (Fig. 1b). The dynamics of each neuron (and, therefore, the sequence of spikes outcoming from the computational element) encodes binary information: sequences synchronized with the autonomous dynamics *ID* of the neurons represent a zero bit, while sequences synchronized with the external reference *R* codify a 1.

## Results

### Static boolean gates

**Figure 2:**
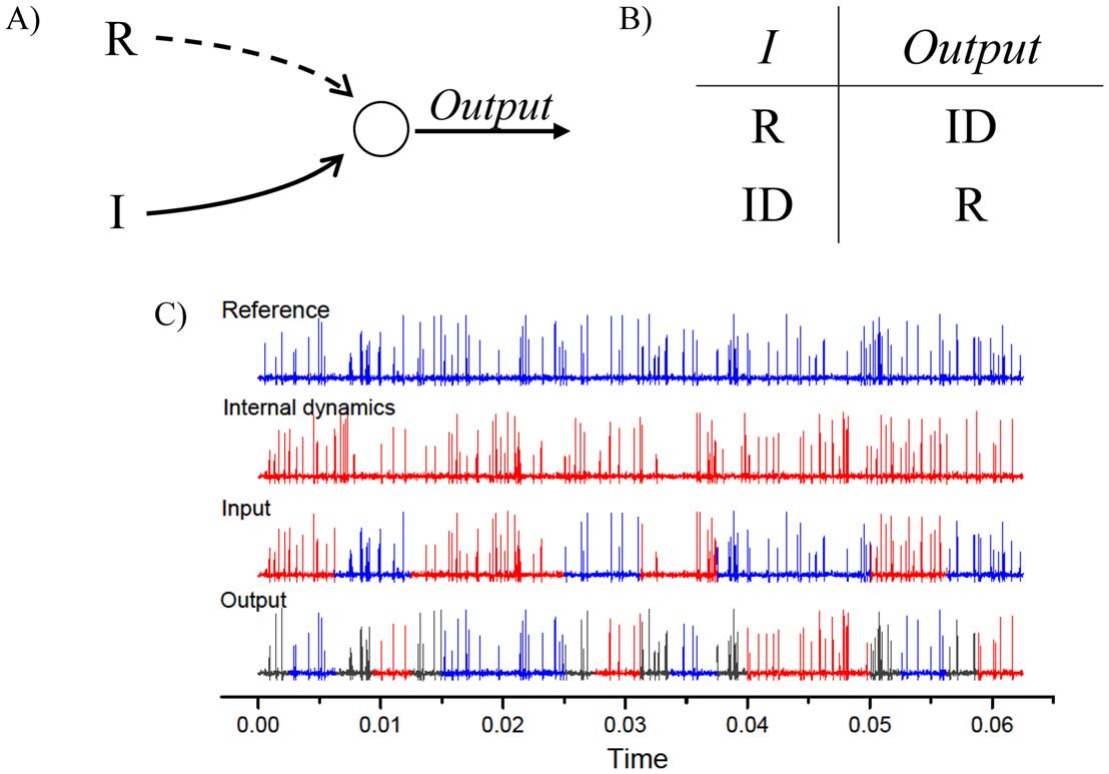
The unary NOT gate. A) The NOT gate is constructed by feeding a reference signal *R* into port *A*, and the signal to be processed into port *B* of the computational unit of Figure 1. B) Following the adaptive dynamics (see the text for further details), the output of the neuron results in the logical opposite of the input. C) (Color online) Temporal snapshots of all signals involved in the computation: the spikes' patterns corresponding to the reference *R*, the internal dynamics *ID* induced by the common noise, and the input and output signals. Following the definition used for the association of the Boolean states (see text), the red (blue) color indicates a 0 (1) bit. The black color is, instead used for coloring the transient intervals of the output signal before its convergence to the logical response.

Using the previously described computational unit, the simplest Boolean operation that can be constructed is the unary NOT gate: a logical gate which returns zero when the input is one, and one otherwise. Such a Boolean gate corresponds to a configuration where the input signal to be processed is fed inside port *B*, while port *A* receives the reference signal *R* (see Fig. 2a). It is straightforward to check that the proposed computational unit performs a NOT operation. An input of 0 is represented by a pattern of spikes synchronized with the autonomous dynamics of the neuron (as e.g. the output of another neuron of the network subjected to the same Gaussian white noise source), thus the error with the signal entering *A* will be greater than zero, and, following the adaptive coupling process, the output will synchronize with the input of *A*, i.e., the 1 reference signal. On the other hand, if the input follows the reference signal *R* (i.e., it is a 1 bit), the synchronization error will be close to zero, thus the output will be the internal dynamics *ID* of the neuron (Fig. 2b). Fig. 2c depicts a temporal snapshot of the spikes' pattern of all the involved signals: *ID*, *R*, and the input and output of the Boolean gate.

**Figure 3:**
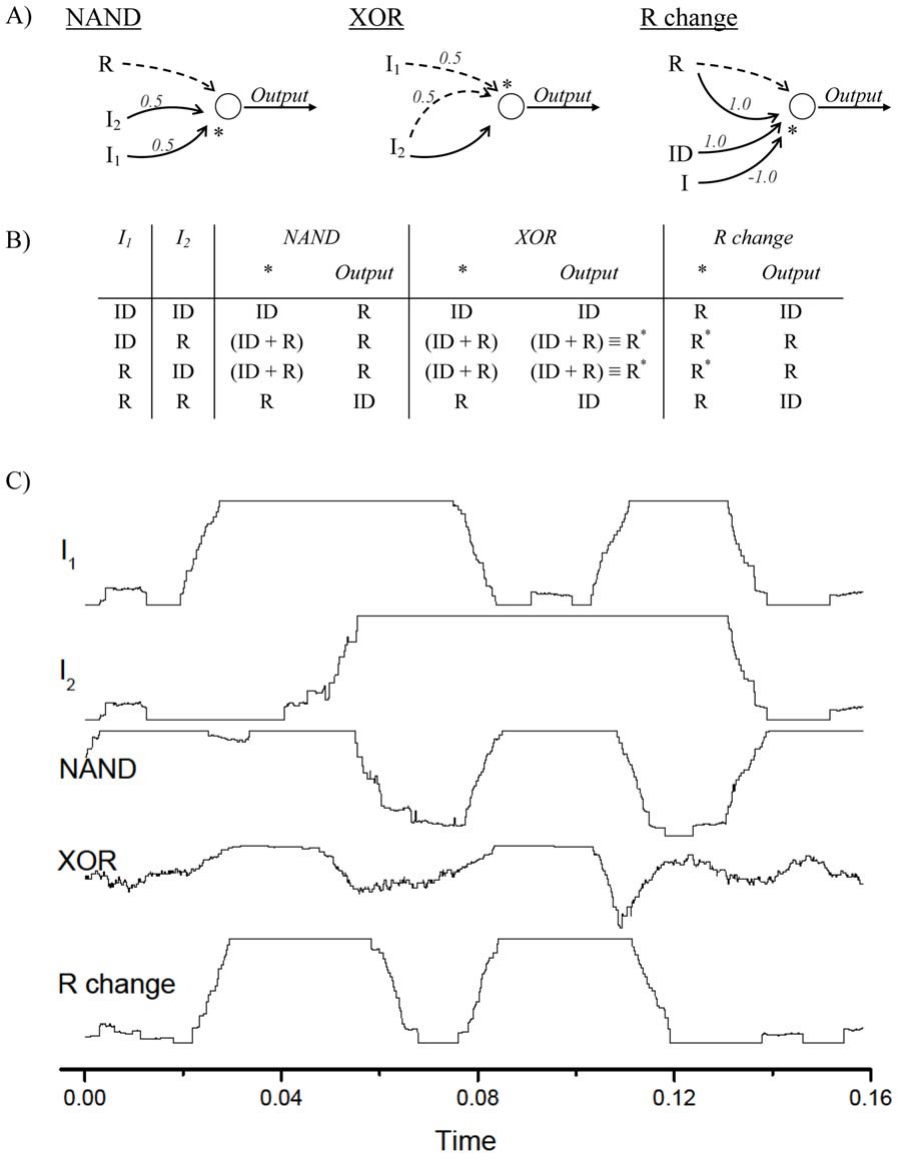
Other binary logical gates. A) Circuit configurations corresponding to the Boolean gates NAND, XOR, and a reference translator (*R change*). Dashed (solid) lines indicate the signal entering port *A* (*B*). In all cases, the numbers indicate the weights of the signals to be summed. B) Table representing the two inputs, the signals present at position * of A), and the output signals. Notice that the output of the XOR operation is expressed in terms of 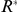, and that the reference translator converts it back to a *R* referenced signal. C) Temporal evolution of 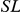 (the synchronization level with the reference signal *R*, see Eq. 5) for the two inputs, and for the output of each gate.

By further embedding the basic computational unit inside a network [1], [18], [19], more complicated logical gates can be constructed. The output of a gate can feed the input(s) of one (or multiple) gates, and each gate may receive information from several neurons: therefore, a circuit can be naturally seen as a weighted network [1]. Several examples are reproduced in Fig. 3. The first is a NAND gate, whose output is zero only when both inputs are 1. This gate is associated to a motif [20] where the reference signal *R* is fed inside the port *A*, and the two input signals are summed (both of them with weight 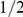) and presented to port *B*. Fig. 3c reports the response of the NAND gate when different inputs are presented; for sake of clarity, the response is represented by the synchronization level SL, defined as:
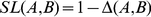
(5)


It is important to remark that the NAND gate is known to be a universal Boolean gate [21], meaning that any Boolean function can be implemented by using a combination of NAND gates, so that any Turing machine can be constructed from them [10]. Following this idea, the second port, the XOR gate (whose output is 1 only when the two inputs have opposite values), can be constructed by joining NAND gates. While this option would require a combination of at least 4 NAND gates, a more efficient strategy is proposed in Fig. 3: port *A* receives the sum of both input signals, each one of them with a weight of 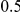, while port *B* only receives the second input. Although this configuration is more efficient, as just a computation element is required, here the output value 1 is no longer encoded by the spike pattern *R*, but by another sequence 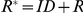. The last gate of Fig. 3 is therefore a configuration that allows switching between different reference signals, i.e., to translate the message: in the example, the output of the XOR gate (expressed in terms of 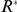) is translated back to *R*. Fig. 3c reports all relevant signals in terms of their synchronization level with *R*, with 1 meaning full synchronization, and 0 no synchrony. Notice that the system is, therefore, not limited to a Boolean coding of the information, with just one representation for each state: multiple reference signals *R* can be used in the same network, thus enabling a richer and more efficient computation.

**Figure 4:**
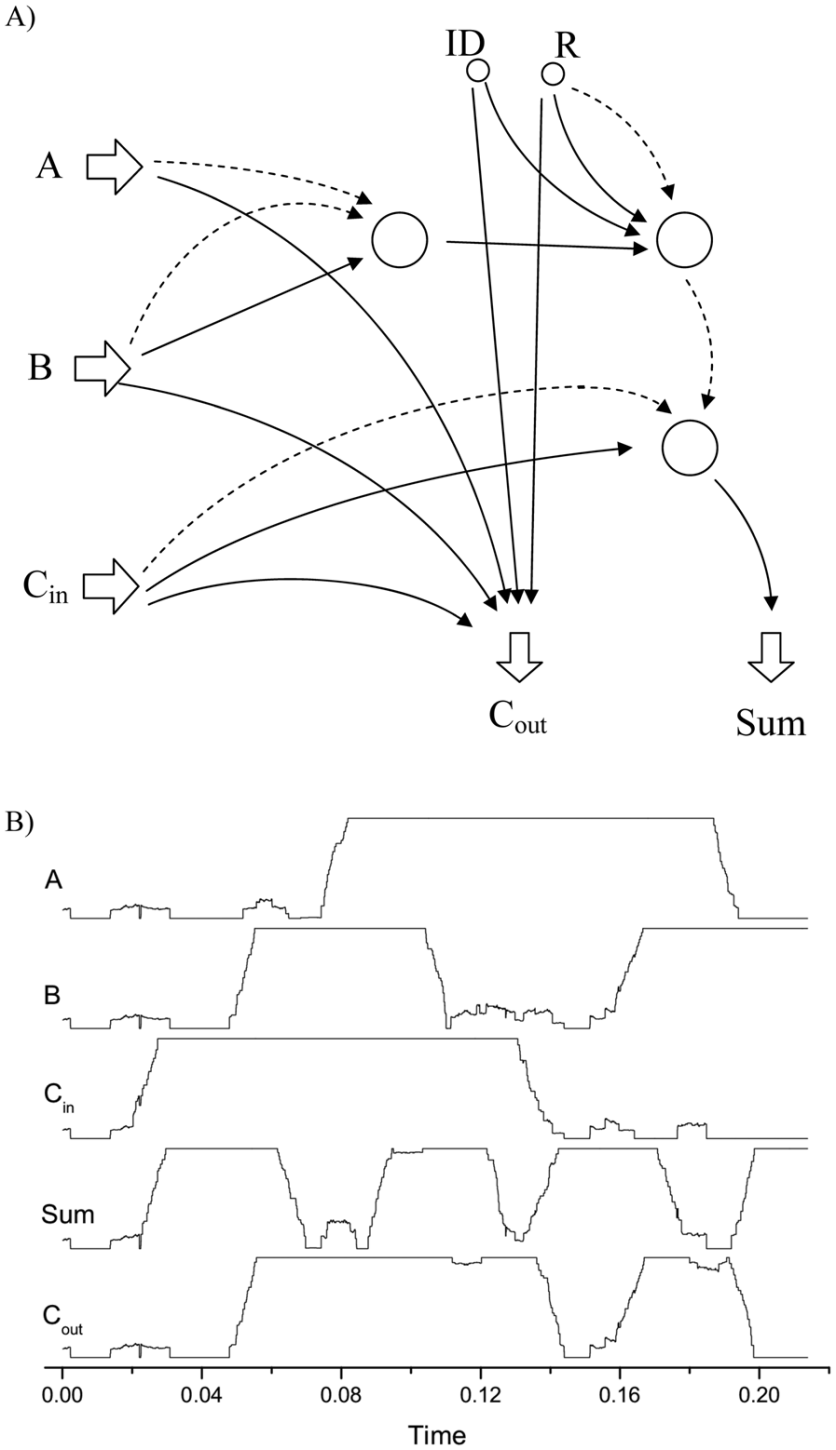
Full boolean adder. A) Network motif corresponding to a full Boolean adder. Same stipulations as for Figure 3a. B) Temporal evolution of 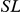 for the three inputs (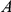, 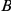 and 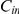) and two outputs (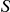 and 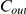) of the adder. The synchronization level 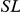 is calculated with respect to the reference signal 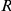 for the three inputs, and with respect to 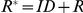 for the outputs.

This feature, that is, the possibility of improving the efficiency of the computation by encoding information with different reference signals, is used in Fig. 4 to create a full Boolean adder. This circuit is composed by three inputs (input bits 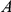 and 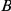, and an input carry 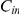) and two outputs: 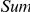 (that is, the sum 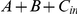) and 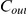 (the carry resulting from the operation 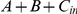). The standard circuit corresponding to this operation would require 15 binary NAND gates [22]; on the other side, the use of the 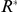 reference signal enables a significant reduction of its complexity, as only 3 computation elements are needed (see Fig. 4a). It should also be noticed that the output 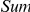 is the result of the activity of three different computation elements, connected in a chain-like motif. This kind of motifs are of interest because of their intrinsic instability: any small perturbation in the input of the system, or any error in the first computation step, may be amplified and result in a wrong output. As can be observed in Fig. 4b, where the numerical simulation of the full binary adder is presented, the proposed computation scheme is stable, even when multiple computation units are used to construct complex circuits.

### Dynamical operations

**Figure 5:**
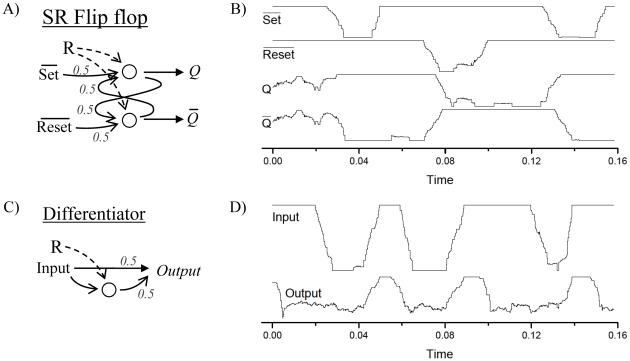
Dynamical logical gates. A) Network motif corresponding to a Set Reset Flip Flop. Same stipulations as for Figure 3a. B) Temporal evolution of 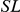 for the two inputs and two outputs of the Flip Flop motif. C) Network motif corresponding to a differentiator gate, whose output is zero all the time, except (and during a limited amount of time) when the input changes from zero to one. D) Temporal evolution of 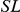 for the input and the output resulting from the differentiation.

Beside the standard, static binary gates, a wider class of dynamical logical operations can also be efficiently constructed, that are of biological relevance. In Fig. 5a we configure a Set Reset Flip Flop circuit. This circuit is a simple memory, composed by two computation units, with two inputs (Set and Reset) and two outputs (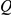 and 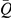). Both units receive the reference *R* on their port *A*, and the weighted sum of an input (*Set* or *Reset*) and the output of the other unit on port *B*. In such a configuration, as long as both input signals are 1, the output is maintained in a stable state by the two loops, with being the binary complement of 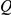; if the set (reset) input changes to 0, the 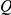 output is forced to 1 (0), and maintain this new status even when both inputs returns to 1. In other words, this circuit acts as a memory, storing a bit and presenting it on its output 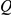, as clearly shown by numerical simulations (Fig. 5b). Likewise the full Boolean adder of Fig. 4a, this motif is also challenging: small errors might be amplified in the two feedback connections, making the system unstable. Nevertheless, the proposed computation unit is resilient against small perturbations and can be used in circuits containing loops, as demonstrated in Fig. 5b.

Finally, Fig. 5c and 5d depict a differentiator, a logical gate whose output is 0 all the time, except when the input changes from level 0 to 1, in which case a positive output is maintained during a fixed amount of time. This simple circuit is configured by feeding *R* on port *A*, the input signal on port *B*, and considering as outcoming signal the weighted sum (with weights 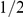) of the output of the computational unit and the input signal itself. The computation here is realized by slowing down the velocity of the adaptive mechanism (see the Methods section for details): when the input changes, the neuron will take some time in updating its coupling forces, thus the input and the neuron's output will be synchronous only within a time window following the change.

## Discussion

In conclusions, we have introduced a basic computation unit, made of a neuron that is interacting with others neurons, following the topology of a dynamical weighted network. Some neurons are forced to synchronize with a reference signal, with a coupling strength that depends on a simple adaptive mechanism, while others are let free to follow their internal dynamics, guided by a common external Gaussian noise. The synchronization and desynchronization of neurons with the reference signal is then used to codify binary information, and we show how the topology of the network is essential for the execution of specific Boolean operations, by means of which it is possible to construct a universal Turing machine.

This approach is of interest for several reasons. First of all, there is a growing quantity of experimental evidences supporting the hypothesis that synchronization is the form used by the brain to represent and compute information [4], [6]. Here we present a first evidence that computation can indeed emerge from the collective dynamics of an ensemble of neurons, without the addition of any external control mechanism. Furthermore, it is important to remark that, being here an emergent feature, the proposed computation is not limited to a binary Boolean logic, but it can be straightforwardly extended to a much larger number of states (by having several reference signals *R*), thus potentially enlightening the real mechanisms at the basis of computation processes of the brain.
